# The histone deacetylase inhibitor suberoylanilide hydroxamic acid attenuates human astrocyte neurotoxicity induced by interferon-γ

**DOI:** 10.1186/1742-2094-9-113

**Published:** 2012-05-30

**Authors:** Andis Klegeris, Patrick L McGeer

**Affiliations:** 1 Kinsmen Laboratory of Neurological Research, Department of Psychiatry, the University of British Columbia, 2255 Wesbrook Mall, Vancouver, BC, V6T 1Z3, Canada; 2 Department of Biology, I.K. Barber School of Arts and Sciences, the University of British Columbia Okanagan, 3333 University Way, Kelowna, BC, V1V 1 V7, Canada; 3 Department of Neuropsychiatry, Graduate School of Medical Sciences, Kyushu University, Maidasi 3-1-1, Higashi-ku, Fukuoka, 812-8582, Japan

**Keywords:** HDAC inhibitor, SAHA, STAT3, I-TAC, Astrocytes, Neuroinflammation, Neurodegenerative diseases

## Abstract

**Backgrounds:**

Increasing evidence shows that the histone deacetylase inhibitor suberoylanilide hydroxamic acid (SAHA) possesses potent anti-inflammatory and immunomodulatory properties. It is tempting to evaluate the potential of SAHA as a therapeutic agent in various neuroinflammatory and neurodegenerative disorders.

**Methods:**

We examined the effects of SAHA on interferon (IFN)-γ-induced neurotoxicity of human astrocytes and on IFN-γ-induced phosphorylation of signal transducer and activator of transcription (STAT) 3 in human astrocytes. We also studied the effects of SAHA on the astrocytic production of two representative IFN-γ-inducible inflammatory molecules, namely IFN-γ-inducible T cell α chemoattractant (I-TAC) and intercellular adhesion molecule-1 (ICAM-1).

**Results:**

SAHA significantly attenuated the toxicity of astrocytes activated by IFN-γ towards SH-SY5Y human neuronal cells. In the IFN-γ-activated astrocytes, SAHA reduced the STAT3 phosphorylation. SAHA also inhibited the IFN-γ-induced astrocytic production of I-TAC, but not ICAM-1. These results indicate that SAHA suppresses IFN-γ-induced neurotoxicity of human astrocytes through inhibition of the STAT3 signaling pathway.

**Conclusion:**

Due to its anti-neurotoxic and anti-inflammatory properties, SAHA appears to have the therapeutic or preventive potential for a wide range of neuroinflammatory disorders associated with activated astrocytes.

## Background

Suberoylanilide hydroxamic acid (SAHA; also known as vorinostat, ChemBank ID 468) is the first histone deacetylase (HDAC) inhibitor approved by the United States Food and Drug Administration. It was licensed in 2006 for the treatment of cutaneous T-cell lymphoma (CTCL) [1]. HDAC inhibitors promote the acetylation of histones, which are generally associated with transcriptional activation. HDAC inhibitors also increase the acetylation status and modulate the activity of a wide range of non-histone proteins. Included are inflammatory transcription factors, such as nuclear factor-κB and signal transducer and activator of transcription (STAT) 3 [1,2]. While various HDAC inhibitors have been studied and developed for cancer therapy due to their anti-proliferative effects, increasing evidence shows that SAHA, at lower and non-cytotoxic concentrations, exhibits potent anti-inflammatory and immunomodulatory activities *in vitro*[3-6] and *in vivo*[4,7]. Furthermore, animal studies indicate that SAHA could ameliorate inflammatory bowel disease [3], hepatitis [4], lupus nephritis [5,6], graft versus host disease [7] and rheumatoid arthritis [8].

A broad spectrum of neurodegenerative diseases, including Alzheimer disease (AD), Huntington disease (HD), Parkinson disease and multiple sclerosis, can be considered as chronic inflammatory disorders of the central nervous system (CNS) [9-11]. Chronic inflammation associated with neuronal damage caused by cerebral ischemia [12] and spinal cord injury [13] could be included. Chronic activation of astrocytes is believed to play an important role in the progression of neuroinflammation, which includes causing damage to the surrounding neurons [10]. The STAT3 signaling pathway has been shown to mediate the neurotoxic secretion of human astrocytes induced by interferon (IFN)-γ [14].

The facts mentioned above motivated us to examine the effects of SAHA on IFN-γ-induced neurotoxicity and STAT3 activation of human astrocytes. The purpose was to evaluate the potential of SAHA as a therapeutic agent in various neuroinflammatory and neurodegenerative disorders. In order to confirm the anti-inflammatory properties of SAHA, we also studied the effects of SAHA on the astrocytic production of two representative IFN-γ-inducible inflammatory molecules, IFN-γ-inducible T cell α chemoattractant (I-TAC) and intercellular adhesion molecule-1 (ICAM-1).

## Methods

### Chemicals and reagents

SAHA was purchased from BioVision (MountainView, CA, USA). Human recombinant IFN-γ was purchased from PeproTech (Rocky Hill, NJ, USA). 3-(4,5-dimethylthiazol-2-yl) 2,5-diphenyl tetrazolium bromide (MTT) and dimethyl sulfoxide (DMSO) were obtained from Sigma-Aldrich (St. Louis, MO, USA). SAHA was initially dissolved in DMSO. The final concentration of DMSO in tissue culture medium was less than 0.001%. At this concentration, DMSO had no effect on cell viability.

### Cell cultures

The human astrocytic U-373 MG cell line was obtained from the American Type Culture Collection (ATCC, Manassas, VA, USA). The human neuroblastoma SH-SY5Y cell line was a gift from Dr. Robert Ross. These cells were grown in Dulbecco’s modified Eagle medium (DMEM), nutrient mixture F12 Ham (DMEM-F12) supplemented with 10% fetal bovine serum (FBS) and penicillin (200 U/ml)/streptomycin (200 μg/ml) (all from Invitrogen Canada, Burlington, ON, Canada). Both cell lines were used without initial differentiation.

Human astrocytes were obtained from epileptic patients undergoing temporal lobe surgery. The specimens were from normal tissue overlying the epileptic foci. The use of human brain materials was approved by the Clinical Research Ethics Board for Human Subjects of the University of British Columbia. Astrocytes were isolated as described previously [15,16]. They were grown in DMEM-F12 supplemented with 10% FBS and penicillin/streptomycin. The cells were cultured for three to four weeks. Purity of the astrocyte cultures was estimated by immunostaining with an antibody against the astrocytic marker glial fibrillary acidic protein (GFAP, from Dako, Z334, Carpinteria, CA, USA). Under our culture conditions, more than 99% cells were positive for GFAP.

### Cytotoxicity of human astrocytes and U-373 MG cells towards SH-SY5Y cells

Human astrocytes or astrocytic U-373 MG cells were seeded into 24-well plates at a concentration of 2 × 10^5^ cells/ml in 0.8 ml of DMEM-F12 medium containing 5% FBS. The cells were treated with various drugs for 1 h prior to the addition of activating stimulant (50 U/ml of IFN-γ). The cells in the control group were incubated with medium only. After 24 h incubation of U-373 MG cells or 48 h incubation of astrocytes at 37°C, 0.4 ml of cell-free supernatants were transferred to each well containing SH-SY5Y cells. At this time point, viability of U-373 MG or astrocytes was measured by the MTT assay. SH-SY5Y cells had been plated 24 h earlier at a concentration of 2 × 10^5^ cells/ml in 0.4 ml of DMEM-F12 medium containing 5% FBS. After 72 h incubation at 37°C, evaluation of surviving SH-SY5Y cells was performed by the MTT assay. The neuronal culture media were sampled for lactate dehydrogenase (LDH) to determine its release from dead cells. To establish that SAHA at the concentration, which showed anti-neurotoxic effects, did not neutralize neurotoxins in the supernatants, the following procedures were used. Supernatants from astrocytes treated with IFN-γ for 48 h in the absence of the drug were collected first. One μM of SAHA was added into the supernatants just before applying them to the SH-SY5Y cells. After 72 h incubation at 37°C, the SH-SY5Y cell viability was measured by the MTT assay. LDH release from dead cells was also measured.

### MTT assay

MTT reduction was measured as described previously [17]. Briefly, the MTT reagent was added to cell cultures to reach a final concentration of 0.5 mg/ml. Following 1 h incubation at 37°C, the dark crystals formed were dissolved by adding to the wells an equal volume of sodium dodecyl sulfate/N, N-dimethylformamide (SDS/DMF) extraction buffer (20% SDS, 50% DMF, pH 4.7). Subsequently, plates were placed overnight at 37°C in order to dissolve aggregates of lysed cells. Optical density (OD) was measured at 570 nm. Viable cell values were expressed as a percentage of the value obtained from cells incubated in fresh medium only. The residual value for 0% cell survival was determined by lysing the cells with 1% Triton X-100.

### LDH assay

LDH activity in supernatants was measured as described previously [17]. Briefly, 100 μl of cell culture supernatants were transferred into the wells of 96-well plates, followed by the addition of 15 μl of lactate solution (36 mg/ml in phosphate-buffered saline (PBS)) and 15 μl of p-iodonitrotetrazolium violet solution (2 mg/ml in PBS). The enzymatic reaction was started by the addition of 15 μl of NAD^+^/diaphorase solution (3 mg/ml NAD^+^; 2.3 mg solid/ml diaphorase). OD was measured at 490 nm. The amount of LDH that had been released was expressed as a fraction of the value obtained in comparative wells where the remaining cells were completely lysed by 1% Triton X-100.

### Analysis of cellular morphology

In order to analyze the morphological changes of SH-SY5Y cells, the cultures were observed with an inverted phase-contrast microscope (Axiovert 200, Carl Zeiss, Oberkochen, Germany) and photographed with a digital camera (Retiga 1300, Qimaging, Surrey, BC, Canada) 72 h after transfer of supernatants from astrocytes. 40x and 20x objectives were used.

### Western blot analysis

Total protein was extracted from subconfluent human astrocyte cultures in 10 cm culture dishes. Astrocytes were incubated with or without SAHA for 1 h followed by incubation with 50 U/ml of IFN-γ for a further 30 minutes. Astrocytes in the control group were incubated with medium only. The cells were washed twice with PBS and then fixed with 10% trichloroacetic acid for 30 minutes at 4°C. Subsequently, the cells were scraped and lysed in ice-cold RIPA buffer (50 mM Tris–HCl (pH 8.0), 150 mM NaCl, 1% deoxycholic acid, 1% TritonX100, 0.1% SDS) supplemented with complete protease inhibitor cocktail (Roche Diagnostics, Mannheim, Germany). The lysed cells were sonicated and then centrifuged at 13,000 *g* for 5 minutes at 4°C and the supernatants were collected. Two μg of protein were subjected to SDS-polyacrylamide gel electrophoresis using an 8% acrylamide gel at 120 V for 70 minutes. The protein was transferred to a PVDF membrane at 70 V for 2 h. The membrane was blocked with 5% skim milk plus 3% bovine serum albumin (BSA) in PBS at room temperature (RT) for 1 h. Subsequently, the membrane was incubated with specific rabbit antibodies against phospho-Tyr^701^-STAT1 (1:2,000), total STAT1 (1:1,000), phospho-Tyr^705^-STAT3 (1:2,000) or total STAT3 (1:1,000) at 4°C overnight and then treated with horseradish peroxidase-conjugated anti-rabbit IgG antibody (1:2,000) at RT for 1 h. All antibodies used for immunoblotting were purchased from Cell Signaling Technology (Danvers, MA, USA). Blots were developed by the chemiluminescent ECL system (Amersham, GE Healthcare, Buckinghamshire, UK). The band intensity was quantified by densitometry using the NIH Image analysis software version 1.63 (NIH, Bethesda, MD, USA). Individual expression level of phosphorylated STAT1 or STAT3 was normalized to the corresponding level of total protein.

### Measurement of I-TAC production: enzyme-linked immunosorbent assay (ELISA)

Human astrocytes were seeded into 48-well plates at a concentration of 2 x 10^5^ cells/ml in 0.4 ml of DMEM-F12 medium containing 5% FBS. The cells were incubated in the presence or absence of SAHA for 1 h prior to the addition of activating stimulant (50 U/ml of IFN-γ). Astrocytes in the control group were incubated with medium only. After 48 h incubation at 37°C, 100 μl of cell-free supernatants were assayed for I-TAC accumulation. The concentrations of I-TAC were measured with an ELISA development kit supplied by PeproTech. The assay was carried out according to the protocol supplied by the manufacturer.

### Measurement of ICAM-1 expression

Human astrocytes were seeded into 48-well plates at a concentration of 2 x 10^5^ cells/ml in 0.4 ml of DMEM-F12 medium containing 5% FBS. The cells were incubated in the presence or absence of SAHA for 1 h prior to the addition of activating stimulant (50 U/ml of IFN-γ). Astrocytes in the control group were incubated with medium only. After 48 h incubation at 37°C, the cells were fixed in 4% paraformaldehyde at 4°C for 5 minutes and then incubated with PBS containing 0.1% Triton X-100 at RT for 5 minutes. After blocking with 5% BSA in PBS for 1 h at RT, the cells were incubated with monoclonal anti-ICAM-1 antibody (1:1,000; MU326-UC, 1 H4, Biogenex, San Ramon, CA, USA) at RT for 2 h followed by incubation with alkaline phosphatase-conjugated goat anti-mouse IgG (1:3,000; Sigma-Aldrich) at RT for 2 h. After washing with PBS, they were incubated with 1 mg/ml of phosphate substrate (Sigma-Aldrich) in 0.1 M diethanolamine buffer (pH 9.8) at RT for 1 h. Subsequently, OD was measured at 405 nm.

### Statistics

All values are expressed as the means ± standard error of mean (S.E.M.). Comparisons were made with a one-way analysis of variance (ANOVA) followed by the *post hoc* Tukey-Kramer test using StatView 5.0 software (SAS Institute Inc., Cary, USA). The significance was established at a level of *P* <0.05.

## Results

### Effects of SAHA on IFN-γ-induced neurotoxicity of human astrocytes and astrocytoma cells

**Figure 1:**
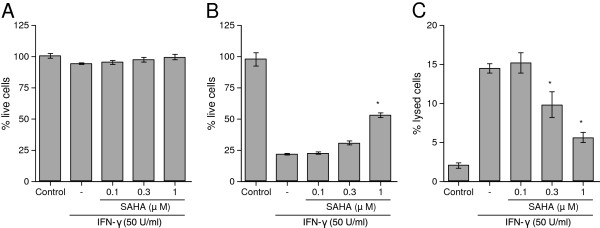
**Effects of SAHA on human astrocytic U-373 MG cell viability and their IFN-γ-induced toxicity toward SH-SY5Y human neuronal cells.** U-373 MG cells were incubated with or without SAHA at the concentrations indicated for 1 h before stimulation with IFN-γ (50 U/ml). Control U-373 MG cells were incubated with medium only. After 24 h incubation, the cell-free supernatants of U-373 MG cells were collected and the viability of U-373 MG was measured by the MTT assay **(A)**. The collected supernatants were transferred to each well containing SH-SY5Y cells. After 72 h incubation, the SH-SY5Y cell viability was assessed by the MTT (B) and the LDH (C) assays. Data (means ± S.E.M.) are expressed as the percent of live cells, where the 100% value is obtained from either non-stimulated astrocytes in the control group (A) or SH-SY5Y cells incubated with fresh medium only **(B)**, or the percent of lysed cells, where the 100% value is obtained from SH-SY5Y cells lysed by 1% Triton X-100 **(C)**. *Significantly different from IFN-γ stimulation only. n = 6 to 7.

We first investigated the effects of SAHA on IFN-γ-induced neurotoxicity of human astrocytic U-373 MG cells. The MTT assay revealed that SAHA did not affect the U-373 MG cell viability in the 0.1 to 1 μM range (Figure 1A). U-373 MG cells caused significant toxicity towards SH-SY5Y cells after 24 h incubation with 50 U/ml of IFN-γ as shown by both the MTT (Figure 1B) and LDH assays (Figure 1C). Pretreatment of U-373 MG cells with 1 μM of SAHA for 1 h significantly prevented the IFN-γ-induced neurotoxicity according to the MTT assay (Figure 1B). The LDH assay also showed significant reduction of the IFN-γ-induced neurotoxicity by SAHA at 0.3 and 1 μM (Figure 1C). In our preliminary studies, we confirmed that 50 U/ml of IFN-γ when added directly to SH-SY5Y cells had no effect on their viability according to the MTT assay (data not shown).

**Figure 2:**
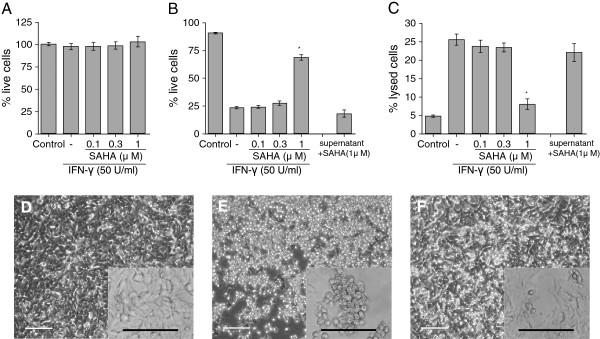
**Effects of SAHA on viability of human primary astrocytes and their IFN-γ-induced toxicity toward SH-SY5Y cells.** Human astrocytes were incubated with or without SAHA at the concentrations indicated for 1 h before stimulation with IFN-γ (50 U/ml) for 48 h (A-F), or SAHA was added directly to cell-free supernatants from IFN-γ-stimulated astrocytes (B, C, right bars). Astrocytes in the control group were incubated with medium only. After 48 h incubation, cell-free supernatants were collected from astrocyte cultures and the astrocyte viability was measured by the MTT assay **(A)**. The collected supernatants were transferred to cultures of SH-SY5Y cells and their viability assessed by the MTT (B) and LDH (C) assays 72 h later. To establish that SAHA does not neutralize neurotoxins in stimulated supernatants, 1 μM of SAHA was added into the supernatants from astrocytes stimulated with IFN-γ (50 U/ml) for 48 h just before applying such supernatants to SH-SY5Y cell cultures. After 72 h incubation, the SH-SY5Y cell viability was assessed by the MTT assay (B, right bar) or the LDH assay (C, right bar). A phase-contrast microscopy with 20x and 40x (see inserts) objectives was used to analyze morphology of SH-SY5Y cells (D-F). D, control; E, IFN-γ alone; F, 1 μM SAHA + IFN-γ. Every scale bar indicates 100 μm (D-F). Data (mean ± S.E.M.) are expressed as percent of live cells, where the 100% value is obtained from either non-stimulated astrocytes in the control group (A) or SH-SY5Y cells incubated with fresh medium only **(B)**, or percent of lysed cells, where the 100% value is obtained from cells lysed by 1% Triton X-100 **(C)**. *Significantly different from IFN-γ stimulation only. n = 6.

We further established the SAHA neuroprotection by using primary human astrocytes. The MTT assay demonstrated that SAHA did not affect the viability of human astrocytes in the 0.1 to 1 μM range (Figure 2A). Human astrocytes caused significant toxicity towards SH-SY5Y cells after 48 h incubation with 50 U/ml of IFN-γ (Figure 2B, C). Similar to the results with U-373 MG cells, 1 μM of SAHA significantly decreased the IFN-γ-induced neurotoxicity of human astrocytes (Figure 2B, C). To establish that SAHA acts directly on astrocytes and to rule out the possibility that it neutralizes neurotoxins, we collected supernatants from astrocytes that had been stimulated with IFN-γ for 48 h without any drug treatment. We then added 1 μM of SAHA into the supernatants just before applying them to SH-SY5Y cells. Addition of 1 μM SAHA did not affect the SH-SY5Y cell viability compared with supernatants without such additions (Figure 2B, C, right bars), suggesting that SAHA does not act by neutralizing neurotoxins following their secretion into the supernatants.

The morphology of SH-SY5Y cells incubated in supernatants of human astrocytes was also analyzed. The supernatants of astrocytes stimulated with IFN-γ caused significant changes in cellular morphology (Figure 2E). The majority of the cells showed bright and circularly shrunk cytoplasm in contrast to the typical healthy morphology presented in the control group (Figure 2D). This change was considerably attenuated by pretreatment with 1 μM SAHA (Figure 2F). These observations were in line with the results obtained by both the MTT and LDH assay (Figure 2B, C).

### Effects of SAHA on IFN-γ-induced phosphorylation of STAT3 in human astrocytes

**Figure 3:**
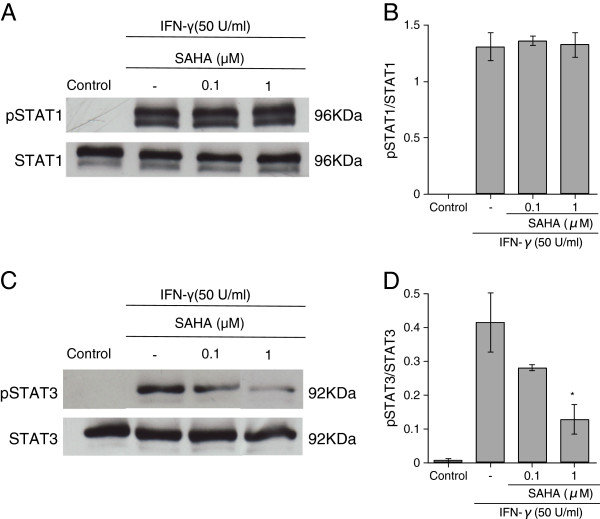
**Effects of SAHA on IFN-γ-induced phosphorylation of STAT1 and STAT3 in human astrocytes.** Human astrocytes were incubated with or without SAHA at the concentrations indicated for 1 h. The cells were subsequently stimulated with IFN-γ for 30 minutes. Astrocytes in the control group were incubated with medium only. Cell lysates were separated by 8% SDS-PAGE and immunoblotted for phospho-Tyr^701^-STAT1 (pSTAT1) and total STAT1 **(A)** or phospho-Tyr^705^-STAT3 (pSTAT3) and total STAT3 **(C)**. The density ratios of phosphorylated to total protein are shown as mean ± S.E.M. of three independent experiments **(B, D)**. *Significantly different from IFN-γ stimulation only.

Our recent studies have indicated that STAT3 signaling, but not STAT1 signaling, mediates IFN-γ-induced neurotoxicity of human astrocytes [14]. Therefore, we investigated the effects of SAHA on the IFN-γ-induced phosphorylation of Tyr^701^-STAT1 and Tyr^705^-STAT3 in human astrocytes. Treatment of astrocytes with 50 U/ml of IFN-γ for 30 minutes phosphorylated both Tyr^701^-STAT1 (Figure 3A) and Tyr^705^-STAT3 (Figure 3C). Densitometry revealed that 1 h pretreatment with 1 μM of SAHA significantly inhibited the STAT3 phosphorylation (Figure 3D), while the drug did not affect the STAT1 phosphorylation (Figure 3B). These results suggest that SAHA reduces IFN-γ-induced neurotoxicity of human astrocytes via inhibition of STAT3 phosphorylation.

### Effects of SAHA on IFN-γ-induced I-TAC production and ICAM-1 expression by human astrocytes

**Figure 4:**
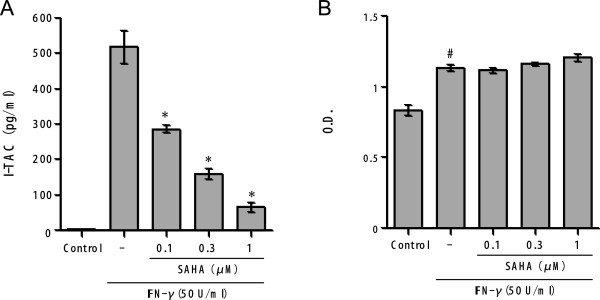
**Effects of SAHA on I-TAC production and ICAM-1 expression by IFN-γ-activated human astrocytes.** Human astrocytes were pretreated with or without SAHA at the concentrations indicated for 1 h before stimulation with IFN-γ (50 U/ml). Astrocytes in the control group were incubated with medium only. After 48 h incubation, I-TAC concentrations were determined in the cell-free supernatants of astrocytes **(A)** and the astrocytic expression of ICAM-1 was measured **(B)**. *Significantly different from IFN-γ stimulation only. ^#^Significantly different from unstimulated control. n = 7. O.D., optical density units.

We finally examined the effect of SAHA on production of the inflammatory chemokine I-TAC and on expression of the inflammatory adhesion molecule ICAM-1 by human astrocytes stimulated with IFN-γ. Incubation of astrocytes with 50 U/ml of IFN-γ for 48 h significantly increased the I-TAC production (Figure 4A) and ICAM-1 expression (Figure 4B). SAHA significantly reduced the IFN-γ-induced I-TAC production in a concentration-dependent manner (Figure 4A). SAHA, in the same concentration range, did not suppress the IFN-γ-induced ICAM-1 expression (Figure 4B).

## Discussion

There were three major findings in the present study. First, SAHA significantly reduced the IFN-γ-induced neurotoxicity of human astrocytes and U-373 MG cells at non-cytotoxic concentrations. Second, SAHA inhibited the phosphorylation of Tyr^705^-STAT3 in human astrocytes stimulated with IFN-γ. Third, SAHA significantly suppressed the I-TAC production, but not ICAM-1 expression, by IFN-γ-activated human astrocytes.

The inhibitory effect of SAHA on human astrocyte neurotoxicity is compatible with emerging data from several *in vitro* studies using various stimulated immune cells, which show anti-inflammatory properties of SAHA. Specifically, treatment of SAHA has been shown to down-regulate the cellular production of inflammatory mediators, such as tumor necrosis factor (TNF)-α, interleukin (IL)-1β, IL-6, IL-12, IFN-γ and nitric oxide (NO), which are all potentially neurotoxic [3-6,18]. Moreover, the anti-inflammatory activities of SAHA have also been established *in vivo*. Administration of SAHA has been demonstrated to reduce serum levels of pro-inflammatory cytokines, including TNF-α, IL-1β and IFN-γ, in mice injected with lipopolysaccharide (LPS) [4] or mice transplanted with allogenic bone marrow [7,18]. Therefore, SAHA appears to have therapeutic or preventive potential for a wide range of neuroinflammatory and neurodegenerative disorders. In fact, recent preclinical studies have shown that SAHA administration rescues cognitive deficits in the APPswe/PS1dE9 transgenic mouse model of AD [19] and that SAHA administration improves motor impairments in the R6/2 transgenic mouse model of HD [20]. SAHA has been also demonstrated to decrease ischemic injury in the mouse brain subjected to middle cerebral artery occlusion [21].

Reduction of IFN-γ-induced STAT3 phosphorylation in human astrocytes by SAHA is consistent with recent *in vitro* studies, which showed that SAHA treatment decreased STAT3 phosphorylation in human CTCL HuT78 cells transfected with a STAT3-specific reporter construct [22] and in murine splenocytes stimulated with LPS [18]. Furthermore, HDAC inhibitors other than SAHA, such as trichostatin A (TSA) [23] and AR-42 [24], have also been reported to suppress STAT3 phosphorylation in various cancer cells. On the other hand, it was reported that SAHA did not affect protein levels of phosphorylated STAT3 in HuT78 cells [25,26]. We currently have no clear rationale for the discrepancy and consider that the effects of HDAC inhibition on the intracellular STAT3 phosphorylation remain inconclusive. Nevertheless, histone acetylation induced by HDAC inhibitors may reduce STAT3 phosphorylation by up-regulating expression of suppressors of cytokine signaling (SOCS) 1 and SOCS3, which are negative regulators of the Janus kinase/STAT signaling, as demonstrated by Xiong *et al*. (2012) [23].

The finding that SAHA attenuates both IFN-γ-induced neurotoxicity and IFN-γ-induced STAT3 phosphorylation of human astrocytes is in line with our recent study which demonstrated that IFN-γ-induced neurotoxicity of human astrocytes is mediated, at least in part, by the STAT3 signaling pathway [14]. Proton pump inhibitors [27] and L-type calcium channel blockers [28] have also been demonstrated to suppress IFN-γ-induced astrocytic neurotoxicity and STAT3 activation in human astrocytes. These emerging data further support our hypothesis that neuroprotective activity of many reagents that reduce IFN-γ-induced neurotoxicity of human astrocytes is exerted through inhibition of the STAT3 signaling pathway in these cells.

HDAC inhibitors have been shown to confer neuroprotection in experimental models of various neurodegenerative diseases, including HD [29], amyotrophic lateral sclerosis [30] and multiple sclerosis [31], even though the exact mechanisms underlying their neuroprotective actions are still elusive. As we demonstrated in this study using SAHA, inhibition of activated astrocytes by decreasing intracellular STAT3 phosphorylation seems to be one of the mechanisms. Effects of HDAC inhibitors on astrocytes have not been studied well. SAHA is shown to inhibit the increased amount of TNF-α and NO secretion from Abcd1/2-silenced murine astrocytes, which are associated with inflammatory responses [32]. TSA is indicated to alleviate 1-methyl-4-phenylpyridinium-induced impairment of glutamate uptake by rat astrocytes [33]. HDAC inhibitors are also reported to increase gene expression of the neurotrophins glial cell line-derived neurotrophic factor and brain-derived neurotrophic factor in rat astrocytes [34,35]. All these astrocytic events could contribute to the HDAC inhibitor neuroprotection. The exploration of the relationship between HDAC inhibitor-elicited neuroprotection and astrocytic functions affected by HDAC inhibitors appears to be still in its infancy.

To the best of our knowledge, this is the first study to determine the effects of SAHA on the cellular production of I-TAC. Our results showed that SAHA suppressed the IFN-γ-induced astrocytic production of I-TAC, a non-ELR CXC chemokine which attracts activated T cells during immune and inflammatory responses. This finding is in agreement with a number of previous studies, which have established that various HDAC inhibitors exert anti-inflammatory effects via inhibiting levels of chemokines [36-39] as well as pro-inflammatory cytokines [37,40].

We observed no influence of SAHA on the IFN-γ-induced astrocytic expression of ICAM-1, which contrasts the data obtained by Takada *et al*. (2006) [41] demonstrating that SAHA represses the levels of ICAM-1 expressed by KBM-5 human myeloid cells stimulated with TNF-α. Further studies exploring this discrepancy are clearly warranted.

SAHA may be suitable for a clinical intervention targeting the CNS due to its safety and permeability across the blood–brain barrier (BBB). SAHA is generally well tolerated in clinical trials involving lymphoma patients [1,2] and is reported to cross the BBB and cause biological responses in the mouse brain [20]. The above observations combined with the major findings of the present study identify SAHA as an excellent candidate drug for preclinical testing in a wide range of neuroinflammatory disorders associated with activated astrocytes.

## Abbreviations

AD, Alzheimer disease; BBB, Blood–brain barrier; CNS, Central nervous system; CTCL, Cutaneous T-cell lymphoma; ELISA, Enzyme-linked immunosorbent assay; GFAP, Glial fibrillary acidic protein; HD, Huntington disease; HDAC, Histone deacetylase; ICAM-1, Intercellular adhesion molecule-1; IFN, Interferon; I-TAC, IFN-γ-inducible T cell α chemoattractant; IL, Interleukin; LDH, Lactate dehydrogenase; LPS, Lipopolysaccharide; MTT, 3-(4,5-dimethylthiazol-2-yl) 2,5-diphenyl tetrazolium bromide; NO, Nitric oxide; SAHA, Suberoylanilide hydroxamic acid; SOCS, Suppressors of cytokine signaling; STAT, Signal transducer and activator of transcription; TNF, Tumor necrosis factor; TSA, Trichostatin A.

## Competing interests

Authors declare that they have no competing interests.

## Authors’ contributions

SH and PLM participated in the design of the study. SH carried out all experiments, collected the data and performed the statistical analysis. SH and AK interpreted the data. SH drafted the manuscript. AK and PLM revised the manuscript. All authors read and approved the final manuscript.
